# Quasi Similar Routes of NO_2_ and NO Sensing by Nanocrystalline WO_3_: Evidence by In Situ DRIFT Spectroscopy

**DOI:** 10.3390/s19153405

**Published:** 2019-08-03

**Authors:** Lili Yang, Artem Marikutsa, Marina Rumyantseva, Elizaveta Konstantinova, Nikolay Khmelevsky, Alexander Gaskov

**Affiliations:** 1 Chemistry Department, Moscow State University, Moscow 119991, Russia; 2 Faculty of Materials Science, Moscow State University, Moscow 119991, Russia; 3 Department of Physics, Moscow State University, Moscow 119991, Russia; 4 LISM, Moscow State Technological University Stankin, 127055 Moscow, Russia

**Keywords:** tungsten oxide, nitric oxide, nitrogen dioxide, gas sensor, paramagnetic sites, DRIFT spectroscopy

## Abstract

Tungsten oxide is a renowned material for resistive type gas sensors with high sensitivity to nitrogen oxides. Most studies have been focused on sensing applications of WO_3_ for the detection of NO_2_ and a sensing mechanism has been established. However, less is known about NO sensing routes. There is disagreement on whether NO is detected as an oxidizing or reducing gas, due to the ambivalent redox behavior of nitric oxide. In this work, nanocrystalline WO_3_ with different particle size was synthesized by aqueous deposition of tungstic acid and heat treatment. A high sensitivity to NO_2_ and NO and low cross-sensitivities to interfering gases were established by DC-resistance measurements of WO_3_ sensors. Both nitrogen oxides were detected as the oxidizing gases. Sensor signals increased with the decrease of WO_3_ particle size and had similar dependence on temperature and humidity. By means of in situ infrared (DRIFT) spectroscopy similar interaction routes of NO_2_ and NO with the surface of tungsten oxide were unveiled. Analysis of the effect of reaction conditions on sensor signals and infrared spectra led to the conclusion that the interaction of WO_3_ surface with NO was independent of gas-phase oxidation to NO_2_.

## 1. Introduction

Nitrogen oxides (NO_x_) are toxic atmospheric pollutants produced by fuel combustion in industry and diesel engines. The main dangers of nitric oxide come from its facile oxidation to the highly toxic NO_2_ gas, as well as the presence of nitric oxide (NO) in photochemical smog and its role in the formation of ground-level ozone [1,2,3,4,5]. In addition, nitric oxide is a biologically important compound produced within living cells. For humans, monitoring the biomarker NO molecules in exhaled breath is a promising approach for non-invasive diagnostics of asthma and other respiratory diseases [6,7].

Since nitrogen oxides have prominent redox activity, resistive-type sensors based on semiconductor metal oxides (SMOx) are suitable for sensing NO_x_. A wide range of sensor materials was elaborated with advantageous sensing behavior to NO_2_, including tin dioxide, indium oxide, and composites with noble metals and organic-inorganic perovskites [8,9]. Tungsten oxide has been established as one of the most sensitive materials for NO_2_ sensors [1,2,10,11]. Numerous works demonstrate the advantageous sensitivity and selectivity of WO_3_-based sensors to NO [2,6,12,13,14]. WO_3_ is an *n*-type semiconductor with a bandgap width of 2.62 eV [15]. An intrinsic oxygen deficiency of tungsten oxide that can be expressed as WO_3−δ_ is associated with the partial reduction of W^6+^ cations to W^5+^. The occurrence of such donor states providing loosely bound electrons is favorable for chemisorption of atmospheric oxygen. The formation of chemisorbed oxygen species (O_2_^−^, O^−^ or O^2−^) was believed to be the initial stage in the sensing mechanism by WO_3_-based sensors [3,5,16]. NO_2_ has stronger electron acceptor behavior than that of oxygen. The standard redox potential of the pair NO_2_/NO_2_^−^ is 0.87 V versus NHE, which exceeds that of O_2_/O_2_^−^ (−0.16 V vs. NHE [17]). The electron affinity of NO_2_ (2.3 eV) is higher than that of O_2_ (0.45 eV) [18]. Thus, chemisorption of NO_2_ on the surface of *n*−type SMOx and electron trapping by the adsorbate is more energetically preferable, than that of the chemisorption of O_2_. Nitrogen dioxide in air is always detected as an oxidizing gas, i.e., by the increase of resistance of n-type SMOx. The sensing mechanism includes oxidation of the WO_3_ surface via the formation of NO_2_^−^ species and fulfillment of oxygen vacancies through the dissociative chemisorption of NO_2_ [19].

The explanation of sensing behavior to NO faces the issue of the attribution of this gas to a particular redox group. In most works, NO was detected as an oxidizing gas by various *n*-type SMOx [3,6,12,14,20,21,22,23,24,25]. This is explained by negative charge accumulation on the solid surface due to NO chemisorption in the form of molecular ions (NO^−^) and/or in the form of residuals of its dissociation into O^−^ and N_2_ [2,3,6,22,23,25]. An alternate explanation is that NO is readily oxidized to NO_2_ in oxygen-containing ambient, and the latter is detected by the sensors as the oxidizing gas [24,26]. However, it has been emphasized that despite the thermodynamic feasibility of NO oxidation to NO_2_ by O_2_, this process is kinetically hampered [27]. For this reason, the sensors that realized the principle of NO to NO_2_ conversion were functionalized by catalytic additives [24,27]. There are some reports on NO being detected as a reducing gas by sensors based on pristine WO_3_ [27] and WO_3_-based composites [13]. The standard NO/NO^−^ redox potential (−0.76 V vs. NHE) is lower than that of O_2_/O_2_^−^ (−0.16 V vs. NHE) [17]. The electron affinity of NO (0.03 eV) is lower than that of O_2_ (0.45 eV) [18]. Thus, NO has weaker electron acceptor behavior than that of O_2_, and it is unlikely that NO can replace oxygen in the competitive adsorption on *n*-type SMOx. Moreover, thermodynamics of NO oxidation by oxygen to NO_2_ suggest that it is energetically preferable for nitric oxide to be a reducing gas in the reaction with oxygen species O_2_^−^ (and a fortiori O^−^) on the surface of SMOx.

In this study, we focused on a comparative study of NO_2_ and NO sensing pathways using tungsten oxide. Nanocrystalline WO_3_ was synthesized with different particle sizes in the range of 7–35 nm. In the sensing tests, the responses of WO_3_ sensors to NO and NO_2_ with respect to the oxidizing gases were observed. Similar dependencies of sensor signals on temperature and humidity were found in the sensing tests to NO_2_ and NO. The effect of WO_3_ particle size on sensitivity was correlated to the concentration of partially reduced cations W^5+^. Similar interaction routes of WO_3_ with NO and NO_2_ were inferred from the results of in situ infrared (DRIFT) spectroscopy. However, from distinct effects of reaction conditions on DRIFT spectra and the concentrations of target gases on sensitivity it was deduced that NO sensing by WO_3_ was not due to the gas-phase conversion of NO to NO_2_.

## 2. Materials and Methods

The nanocrystalline WO_3_ was obtained by aqueous deposition of tungstic acid followed by thermal decomposition at different temperatures [28]. Nitric acid (7.8 M) was dropwise added to the stirred 16 mM aqueous solution of (NH_4_)_10_H_2_W_12_O_42_ (Sigma−Aldrich, >99%) at 80 °C. The resultant mixture of 10 mM ammonium paratungstate and 3 M HNO_3_ was stirred at 80 °C for 30 min and cooled down to room temperature for 1 h. The obtained yellow precipitate of tungstic acid was centrifuged, washed by deionized water, and dried at 80 °C for 12 h. It was divided into four parts, which were annealed in air for 24 h at different temperatures: 300, 450, 600, and 800 °C.

The X−ray powder diffraction (XRD) was measured using a DRON−3M diffractometer, Cu Kα radiation. Grain size was calculated by the Sherrer equation using full width at half maximum of the peaks (002), (020), (200), (022), (220) of monoclinic WO_3_ phase (ICDD 43−1035). Specific surface area was measured following the nitrogen adsorption Brunauer−Emmett−Teller (BET) method. Single-point measurements at *p(*N_2_) = 0.3 atm were performed using the instrument Chemisorb 2750 (Micromeritics). Transmission electron microscopy (TEM) images and electron diffraction (ED) patterns were acquired using a FEI Tecnai G2 microscope operated at 200 kV. The X−ray photoelectron spectra (XPS) were registered with Axis Ultra DLD (Kratos, UK) spectrometer with Al *K*_α_ source. Binding energy was calibrated by C 1s signal at 285.0 eV. The electron paramagnetic resonance (EPR) spectra were recorded at 40 K by the Bruker EPR spectrometer ELEXSYS−580 (X-band, sensitivity is ~10^10^ spin/G). The values of *g*-factors and spin center concentrations were calculated using standard samples Mn^2+^ and CuCl_2_·2H_2_O, respectively.

Diffuse reflectance infrared Fourier transformed (DRIFT) spectra were registered by a Frontier (Perkin Elmer) spectrometer with the DiffusIR annex and flow chamber HC900 (Pike Technologies) sealed by a ZnSe window. The DRIFT spectra were registered in the wavenumber range of 1000–4000 cm^−1^ with a resolution of 4 cm^−1^ and averaging 30 scans at ambient conditions with automatic H_2_O/CO_2_ compensation. The powders (30 mg) were placed in alumina crucibles (5 mm diameter). The DRIFT spectra were registered at room temperature and at 100 °С under an exposure to gas flow (100 mL/min) of NO (20 ppm) or NO_2_ (2 ppm). Before the spectra collection, the samples were heated under pure air flow at 200 °C for 1.5 h to clean the surface from adsorbed humidity.

To perform the sensor test, the samples were dispersed in terpineol and drop deposited on alumina substrates provided with Pt contacts (size 0.3 × 0.2 mm, gap 0.2 mm) and Pt heaters. The sensors were placed in a PC-controlled 4 sensor electrometer equipped with a flow chamber with a volume of 130 cm^3^. Sensors were annealed in air at 300 °C for 14 h to remove the binder. The sensing layer had an area of 1 × 0.5 mm^2^ and a thickness of ~10 μm. The DC resistance was measured at the applied voltage of 1.3 V at a fixed temperature in the range 25–300 °C. The gas flow rate was 100 mL/min. The background and carrier gases were pure air from a pure air generator model “2.0–3.5” (Himelectronica, Moscow, Russia), with impurity concentrations within the limits of 10 ppm H_2_O, 2 ppm CO_2_, and 0.1 ppm hydrocarbons. Certified gas mixtures were used as the source of target gas, NO:N_2_ (101 ± 6 ppm) and NO_2_:N_2_ (21 ± 2 ppm) (Linde Gas Rus, Moscow, Russia). The gas flowrates were controlled by mass flow controllers EL−FLOW (Bronkhorst, AK Ruurlo, The Netherlands). The flows of the pure air and the target gas were mixed with each other using the pipelines and fittings (Camozzi, Brescia, Italy). The humidity of the carrier gas was controlled by mixing two air flows with different flowrates, i.e., the dry one from the generator of pure air, and the humid one purged through a bubbler filled by deionized water. All measurements were performed under steady relative humidity values in the range 0–100% r.h. (room temperature), that was verified using a humidity meter IVTM−7K (Eksis, Moscow, Russia).

## 3. Results

### 3.1. Composition, Microstructure, and Oxidation States of Elements in Samples

**Figure 1:**
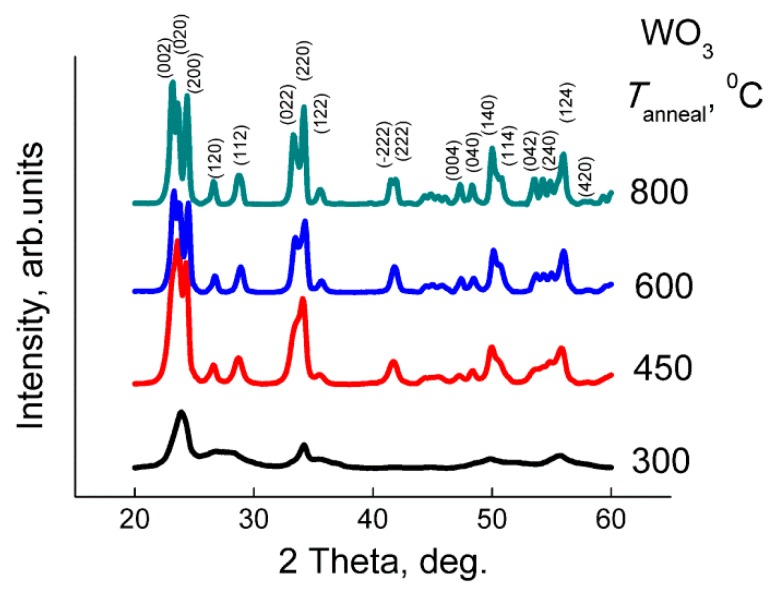
XRD patterns of WO_3_ with different grain size. Peaks are indexed for the monoclinic WO_3_ phase (ICDD 43−1035).

**Figure 2:**
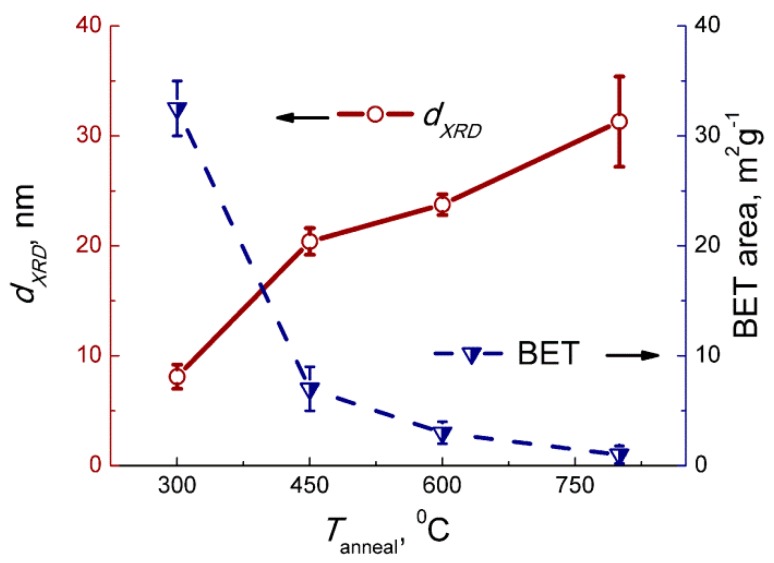
Mean grain size and BET area of WO_3_ in relation to annealing temperature.

**Figure 3:**
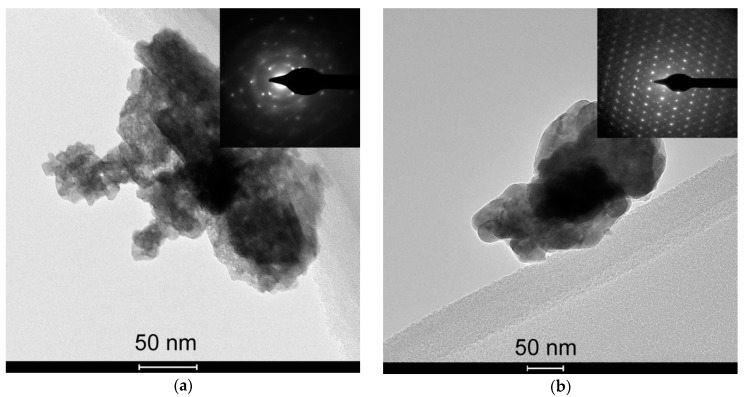
TEM images of WO_3_ samples annealed at 300 °С (**a**) and 450 °С (**b**). The insets show electron diffraction patterns.

According to the XRD patterns (Figure 1), the obtained samples consisted of monoclinic γ-WO_3_ phase (ICDD 43−1035). The average grain size increased, and the BET area decreased, respectively, with the increase of the annealing temperature of WO_3_ (Figure 2). According to the TEM images, the samples are composed of agglomerated WO_3_ nanoparticles (Figure 3). The size of the nanoparticles was 5–10 nm for the sample annealed at 300 °С, and 15–25 nm for the sample annealed at 450 °C, in agreement with the estimations by XRD (7–9 nm and 19–22 nm, respectively). The size of the agglomerates was 50–200 nm. The electron diffraction patterns were not rings, since the nanoparticles were highly oriented in the agglomerates.

**Figure 4:**
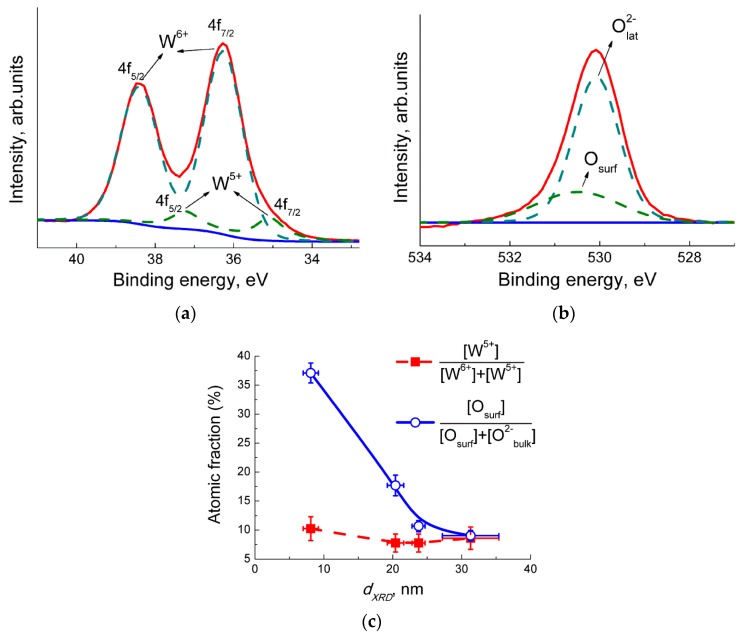
XPS spectra of WO_3_ with grain sizes 7–9 nm in the analytic regions: (**a**) W 4f; (**b**) O 1s. (**c**) Fractions of W^5+^ from total W content and of surface oxygen in total O content in relation to WO_3_ grain size.

By XPS, only tungsten and oxygen were detected in WO_3_ samples. The doublet peaks of W 4f signal are mainly contributed by W^6+^ cations, but a minor contribution from W^5+^ was distinguished (Figure 4a). Oxygen in O 1s spectra was observed in at least two states: the lattice anions O^2−^_lat_ with a binding energy of 530.1 eV, and surface oxygen (O_surf)_ at a higher binding energy of 530.5–531.5 eV (Figure 4b). The surface oxygen was constituted by the anions on the surface of nanocrystals, chemisorbed oxygen, and aqueous species. Atomic fractions of different oxidation states of W and O were estimated from the areas of deconvoluted XPS peaks. The percentage of W^5+^ was 7–11% from the total W content (Figure 4c). The dependence on particle size was insignificant, but within this range the higher concentration of W^5+^ was found in the sample with the least grain size. The fraction of surface oxygen (O_surf_) from the total O content decreased with an increase of WO_3_ grain size (Figure 4c). Such a trend can be explained by the decrease of surface-to-bulk atomic ratio, as well as by lower chemisorption of oxygen and aqueous species on the samples with decreased BET area.

**Figure 5:**
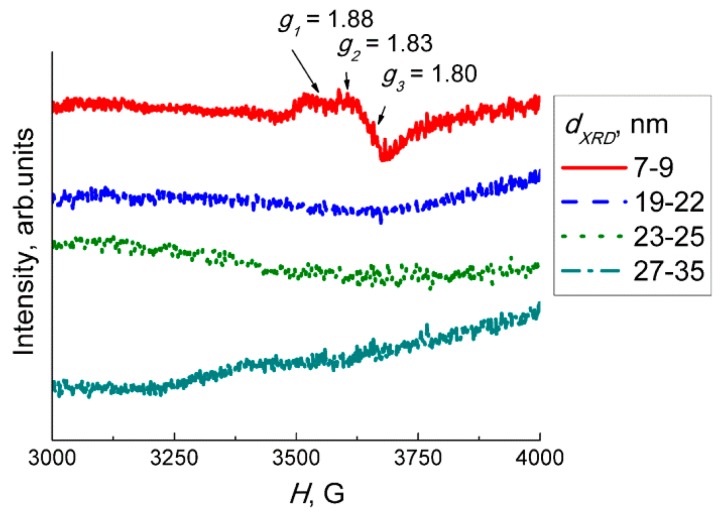
EPR spectra of WO_3_ samples with different grain sizes.

The EPR spectra of WO_3_ samples are shown in Figure 5.) The anisotropic signal with orthorhombic symmetry was detected in the range of magnetic field Δ*H* = 3550–3800 G, only, on the spectrum of the sample with the least grain size (7–9 nm). The g-factor consisted of the following three components: *g*_1_ = 1.88, *g*_2_ = 1.83, and *g*_3_ = 1.80. According to the literature, this EPR signal is due to W^5+^ cations [29]. The concentration of W^5+^ spin centers in this sample was 6 × 10^16^ *g*^−1^, which equals to ~2.3 × 10^−3^% from the total W content. On the spectra of WO_3_ samples with larger particle sizes, this signal is indiscernible from the noise level (Figure 5). More than three orders of magnitude lower percentage of W^5+^ was detected by EPR in WO_3_ with grain sizes 7–9 nm and the absence of W^5+^ in the samples with larger particles is in contrast to the XPS data (Figure 4c). The discrepancy can arise from different measurement conditions: the ultra−high vacuum in XPS setup is favorable for oxygen desorption from WO_3_. The resultant oxygen−deficiency in tungsten oxide WO_3−δ_ accounts for the detection of W^5+^ in all samples by XPS in the over−estimated amounts. On the contrary, EPR spectra were registered under ambient pressure, and the reduced W^5+^ cations were detected only in the WO_3_ with the smallest nanoparticles. Such nanoparticles are more prone to the adsorption-desorption phenomena, including the formation of oxygen vacancies and W^5+^ states.

### 3.2. Sensing Behavior to NO_2_ and NO

**Figure 6:**
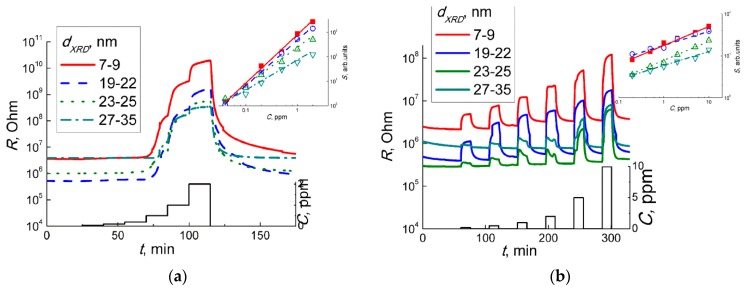
Dynamic responses of WO_3_ sensors to 0.04–2.00 ppm of NO_2_ in air (**a**) and 0.2–10.0 ppm of NO in air (**b**). Insets show sensor signals as functions of the concentrations of target gases in logarithmic axes.

As shown in Figure 6, the dynamic responses of the WO_3_ sensors to different concentrations of NO_2_ (Figure 6a) and NO (Figure 6b) are demonstrated at a temperature of 100 °C. The dynamic responses to nitrogen oxides at different temperatures in the range 25–300 °C are listed in the Supplementary data (Figures S1 and S2). The resistance of the WO_3_ with the smallest grain size (7–9 nm) was ~10 times higher than the resistances of the samples with the larger WO_3_ nanoparticles. The drop in SMOx resistance with an increase of particle size is due to the inferior impact of surface traps to electric conduction. This agrees also with the decrease of specific surface area and surface oxygen species, as shown by XPS in Figure 4**c**. In all cases, the sensor responses to both target gases were observed as to the oxidizing ones, i.e., resistance of WO_3_ increased in the presence of NO_2_ and NO, and it decreased back on exposure to pure air (Figure 6, Figures S1 and S2). Sensor signal (*S*) was defined as the ratio of resistance in the presence of the target gas (*R_g_*) to that in air (*R_a_*):

*S* = *R_g_*/*R_a_* (1)

**Figure 7:**
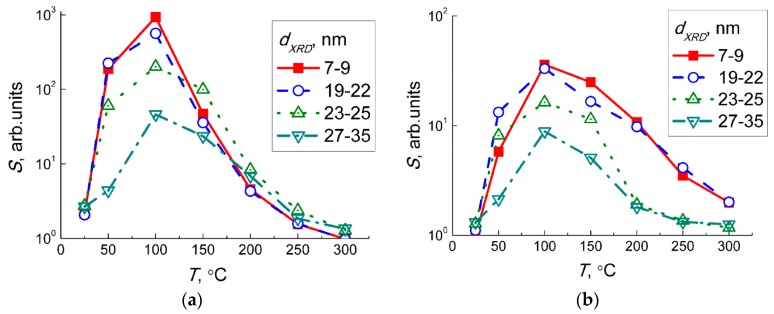
Sensor signals responses of WO_3_ with different grain size to 1 ppm NO_2_ (**a**) and 5 ppm NO (**b**) in relation to temperature.

The sensor signals to fixed concentrations of NO_2_ and NO measured at different temperature are plotted in Figure 7. The sensitivity to both target gases decreased with the increment of WO_3_ particles sizes. Since both NO_2_ and NO behaved as acceptor molecules in the interaction with sensor surface, the effect of particle size can be linked to the occurrence of donor states W^5+^ in the sample with the smallest WO_3_ nanoparticles, as shown by EPR (Figure 5). The drop of sensitivity with increasing grain size may be due to the decrease of specific surface area available for the interaction with NO_x_.

**Figure 8:**
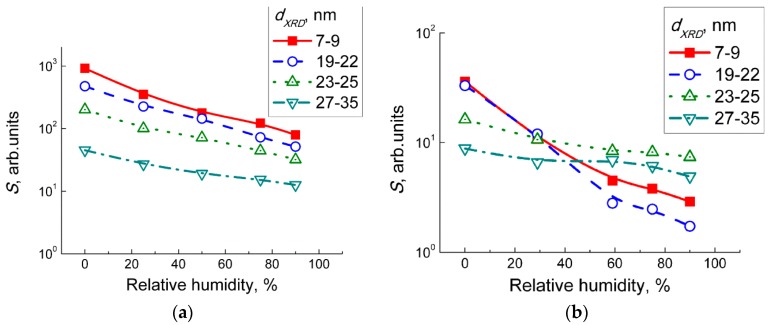
Sensor signals of WO_3_ with different grain size to 1 ppm NO_2_ (**a**) and 5 ppm NO (**b**) at 100 °С and different relative humidity.

The response and recovery times were defined as the periods needed for the sensor to reach 90% of the steady resistance values in target gas (*R_g_*) and in air (*R_a_*), respectively. The response time to NO_2_ in dry air was 6–10 min irrespective of the WO_3_ grain size, operation temperature, and humidity. The recovery time increased from 1–3 min to 9–13 min with a decrease of temperature from 200–300 °С to 100 °С. At room temperature the sensors recovery from NO_2_ was incomplete within a period up to 30 min. Response time to NO was 4–8 min and was unaffected by the WO_3_ particle size, temperature, and humidity. Recovery time after NO exposure was 1–5 min at 200–300 °C. At 100–150 °C, the recovery time was 8–10 min for the sensors with WO_3_ grain sizes 23–35 nm and increased to 15–17 min for sensors with WO_3_ grain size 7–22 nm. The effect of humidity on the sensitivity to NO_2_ and NO was that the sensor signals monotonously decreased as the relative humidity was raised in the range 0–90% r.h. (Figure 8, dynamic sensor responses at different humidity are in Figures S3 and S4 in Supplementary data). However, the sensitivity to NO_x_ was persistent even at 90% relative humidity, which is a promising result with regards to a low operating temperature of 100 °C.

The sensors displayed low cross-sensitivity to other target gases. The sensor signals to a higher concentration of CO, NH_3,_ and CH_4_ and to a comparable concentration of H_2_S were at least one order of magnitude lower than the sensor signals to NO_2_ and NO (Figure S5 in Supplementary data). It is likely that the WO_3_ sensors could selectively discriminate between the oxidizing gases (NO_x_) and the interfering reducing gases under these conditions. The stability of sensors was manifested from the reproducible sensor responses to NO_2_ and NO periodically measured during 2 months (Figure S6 in Supplementary data), providing that, before testing, the sensors were heated at 300 °C in dry air for 2 h to remove the adsorbed humidity and recover the surfaces after previous sensing tests.

### 3.3. DRIFT Study of WO_3_ Interaction with Nitrogen Oxides

**Figure 9:**
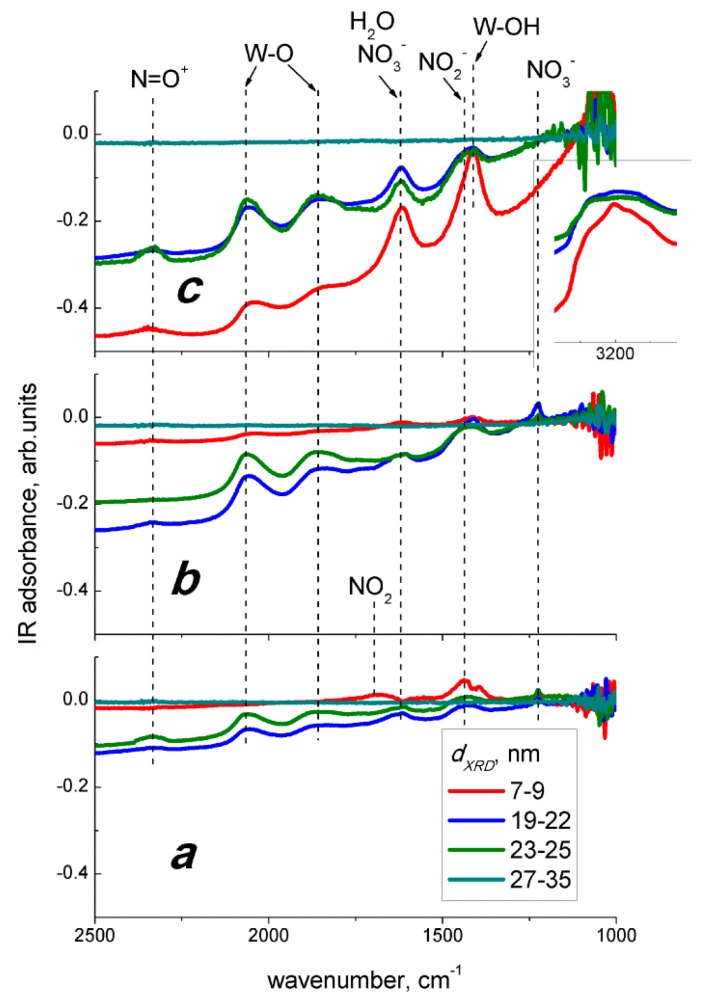
DRIFT spectra of WO_3_ with different grain size exposed to 2 ppm NO_2_ in dry air at 25 °C (**a**), in dry air at 100 °С (**b**), and in 90% r.h. humid air at 100 °С (**c**).

**Figure 10:**
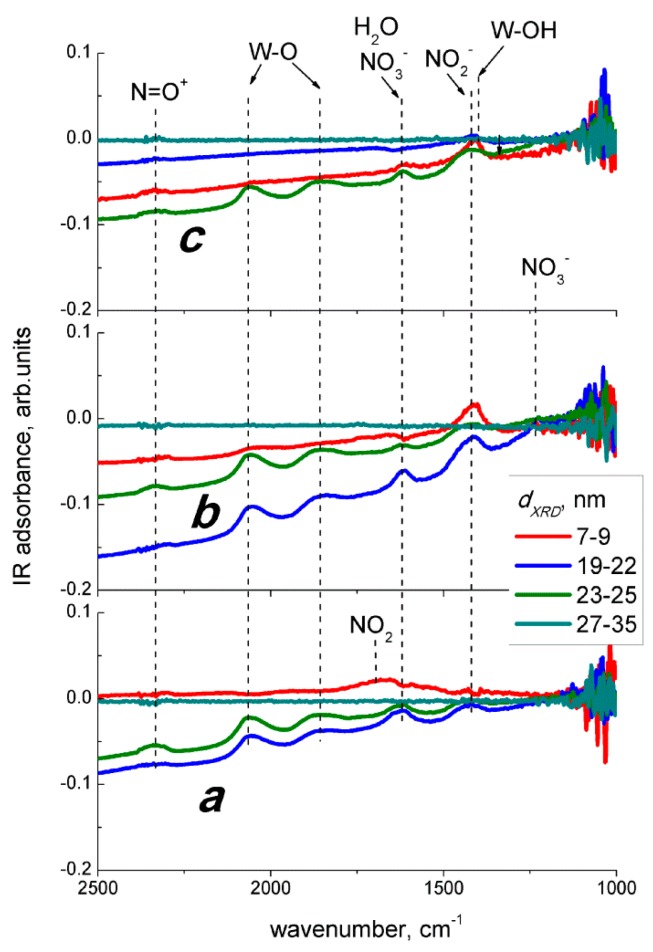
DRIFT spectra of WO_3_ with different grain size exposed to 20 ppm NO in dry air at 25 °C (**a**), in dry air at 100 °С (**b**), and in 90% r.h. humid air at 100 °С (**c**).

Infrared absorption spectra of nanocrystalline tungsten oxide samples have been published in [28]. The bands with stretching vibrations of W = O and the W-O-W bonds and bending vibrations of W-OH bonds were observed in the spectra along with the bands of stretching O-H and bending H_2_O vibrations due to adsorbed humidity [28]. The solid-gas interactions between WO_3_ and nitrogen oxides were studied by DRIFT spectroscopy in the following three modes: (a) adsorption at room temperature, (b) reaction at 100 °C in dry ambient that simulates the optimum sensing conditions, and (c) reaction at 100 °C under 90% relative humidity. The corresponding DRIFT spectra under exposure to 2 ppm NO_2_ are shown in Figure 9, and those under exposure to 20 ppm NO are shown in Figure 10. The common spectral features brought about by the interaction with both nitrogen oxides were the descending background absorption because of the electron depletion of semiconductor grains, and the appearance of the overtone peaks of W-O bonds and the peaks of various oxidized nitrogen species (Figure 9 and Figure 10). It is noteworthy that, no spectral changes were found for the sample with the largest nanoparticles (27–35 nm), which accounts for its lower sensitivity to NO_x_. All the spectral changes observed at 100 °C were reversible. The DRIFT spectra recovered baseline absorption when the samples were evacuated in air for 1.5 h at 100 °С after the exposure to NO_x_.

At room temperature, the adsorption of NO_2_ is followed by the appearance of the band at 1680–1630 cm^−1^ relevant to asymmetric stretching vibrations of adsorbed NO_2_ [30] (Figure 9a). The band at 1450–1390 cm^−1^ can be attributed to stretching N = O vibrations of monodentate nitrite or asymmetric stretching NO_2_ vibrations of the nitro groups [30]. In any case, the NO_2_^−^ species resulted from single electron reduction of adsorbed nitrogen dioxide. The wide low-intense band at 2350–2330 cm^−1^ could be due to N = O vibrations in nitrosonium NO^+^ [30], but in another study it was attributed to an overtone of W-O vibrations [19]. The peak at 1210 cm^−1^ is likely due to stretching vibrations of N-O bonds in monodentate nitrite species. The broad bands at 2060 cm^−1^ and 1880 cm^−1^ are due to overtones of W-O vibrations and reflect the surface oxidation of tungsten oxide via the fulfillment of oxygen vacancies (V_O_) [19].

The main difference in the spectra of NO adsorption on WO_3_ is the absence of the peak of monodentate nitrite at 1210 cm^−1^ (Figure 10a). The band at 1430–1420 cm^−1^ observed on the spectra of WO_3_ with grain size 19–25 nm is likely due to the vibrations of nitro groups and not related to nitrite species. The surface of WO_3_ was also oxidized under these conditions, as inferred from the positive W-O bands at 2060 cm^−1^ and 1880 cm^−1^. The only band observed on the spectrum of WO_3_ with grain size 7–9 nm was that of the adsorbed NO_2_ (1680–1650 cm^−1^).

Similar to room temperature adsorption, the interaction with NO_2_ at 100 °C resulted in positive bands of W-O vibrations at 2060 cm^−1^, 1880 cm^−1^, and/or NO^+^ (2350–2330 cm^−1^) indicating surface oxidation of WO_3_ and probable appearance of NO^+^ (Figure 9b). The band at 1430–1420 cm^−1^ was narrower and shifted to lower wavenumbers as compared with room temperature NO_2_ adsorption, suggesting that it was mainly contributed to by nitro groups NO_2_^−^, rather than by nitrite species [30]. Two bands with comparable intensities and broadenings observed at 1630–1610 cm^−1^ and at 1225–1220 cm^−1^ were assigned to asymmetric and symmetric stretching NO_2_ vibrations of bridging nitrate (NO_3_^−^) species. The interaction of WO_3_ with NO_2_ in humid air was followed by the appearance of the same peaks as in dry air (Figure 9c). Moreover, the spectral changes for the sample with the smallest WO_3_ nanoparticles (7–9 nm) were more pronounced than that of those in dry air. This can be attributed to the larger specific surface area of this sample available for interaction with NO_2_ and humidity. The stronger descent of IR background and the increase of W-O peaks may be due to the cooperative oxidizing effect of humidity and NO_2_ [19]. The adsorption of aqueous species in the presence of NO_2_ on this sample may also be inferred from the intense peaks at 1620 cm^−1^ (bending H_2_O vibrations), 1410 cm^−1^ (bending W−O−H vibrations), and 3220 cm^−1^ (stretching O-H vibrations of hydrogen-bounded hydroxyl groups) (Figure 9c, inset). The traces of bridging nitrates at 1220–1225 cm^−1^, although absent on the spectrum of this sample, were still distinguishable on the spectra of WO_3_ with grain sizes 19–25 nm.

The interaction with NO at 100 °C resulted in different peaks on the spectra of WO_3_ with different particle sizes (Figure 10b). For the sample with the lowest grain size (7–9 nm), the stronger peak was that of the nitro groups (1430–1420 cm^−1^), and the weak bands of adsorbed NO_2_ (1670–1650 cm^−1^), W-O bonds (2060 cm^−1^, 1880 cm^−1^), and/or NO^+^ (2350–2330 cm^−1^) were observed. On the spectra of samples with WO_3_ grain sizes 19–25 nm, the same bands were observed, but the peak of adsorbed NO_2_ was absent. Instead, the peaks of bridging NO_3_^−^ evolved at 1610 cm^−1^ and 1225 cm^−1^. The spectral features in the presence of NO in humid air were much less prominent (Figure 10c) as compared with the interaction with NO in dry air (Figure 10b) or with NO_2_ in dry and humid air (Figure 9b,c). The weak peaks observed in Figure 10c were those at 2350–2330 cm^−1^, 1620 cm^−1^, and 1410 cm^−1^. The peaks of the nitro groups at 1430–1420 cm^−1^ decreased and the peaks of the nitrate species at 1225–1220 cm^−1^ disappeared. The absence of W-O peaks at 2060 cm^−1^ and 1880 cm^−1^ and the presence of the band at 2350–2330 cm^−1^ suggest that the latter was due to NO^+^, rather than W-O overtones. The more prominent peaks attributable to aqueous species H_2_O (1620 cm^−1^), W-OH (1410 cm^−1^) on the spectrum of WO_3_ with grain size 7–9 nm, may be due to the influence of NO oxidation products on water chemisorption.

## 4. Discussion

In this study, we discuss the interaction routes of NO_2_ and NO with the surface of WO_3_ inferred from DRIFT spectra, and we propose the possible sensing mechanisms. Both nitrogen oxides behaved as oxidizing gases relative to WO_3_ under the following sensing test conditions: concentration of NO_x_ in air within the range 0.04–10 ppm, temperature in the range 25–300 °С, and relative humidity in the range 0–90%. The oxidizing behavior of NO_2_ agrees with the higher electron affinity and NO_2_/NO_2_^−^ redox potential as compared to oxygen (the parameters are given in the Introduction). The surface species observed on DRIFT spectra at room temperature (Figure 9a) suggest that the adsorption of NO_2_ on WO_3_ proceeds via the following possible routes:

NO_2(gas)_ = NO_2(ads),_ (2)

NO_2(gas)_ + *e*^−^ = NO_2_^−^_(ads),_ (3)

NO_2_^−^_(ads)_ + V_O(s)_ + W^6+^_(s)_ = NO^+^_(ads)_ + W^6+^−O^2−^_(s),_ (4)

where, *e*^−^ denotes free electron in *n*−type WO_3_ and may be interpreted as an extra electron associated with W^5+^ sites observed by XPS and EPR. The evolution of nitro groups, nitrate species, and surface oxidation of WO_3_ on DRIFT spectra registered at 100 °C (Figure 9b) implied that the interaction with NO_2_ under sensing test conditions proceeded via the reaction (Equations (3) and (4)), along with the disproportionation of NO_2_ or oxidation of NO_2_^−^:

2 NO_2(gas)_ + ½ O_2(gas)_ + 2 *e*^−^ = NO_2_^−^_(ads)_ + NO_3_^−^_(ads),_ (5)

NO_2_^−^_(ads)_ + ½ O_2(gas)_ = NO_3_^−^_(ads)_ (6)

The interaction of WO_3_ with nitrogen dioxide in humid air involved the reactions with NO_2_ according to Equations (3)–(6) and the chemisorption of water, as suggested by the peaks observed on DRIFT spectra in Figure 9c. The fact that the observed peaks intensities were not deteriorated in humid air, accounts for the persistence of WO_3_ sensitivity to NO_2_ in the wide range of humidity (Figure 8). The chemisorption of H_2_O promoted by the presence of NO_2_ is explained by the disproportionation reaction on the surface of WO_3_:

2 NO_2(gas)_ + H_2_O_(ads)_ = 2 H^+^_(surf)_ + NO_2_^−^_(ads)_ + NO_3_^−^_(ads)_. (7)

The trend of temperature dependencies of sensor signals to NO_2_ (Figure 7a) and NO (Figure 7b) coincided, demonstrating maximum sensitivity at about 100 °C. It allows assuming that the sensing mechanisms were similar, or that in both cases the sensor response was due to NO_2_. In the case of NO sensing, some concentration of NO_2_ can result from the gas-phase reaction when nitric oxide was diluted by air:

2 NO_(gas)_ + O_2(gas)_ = 2 NO_2(gas)._ (8)

**Figure 11:**
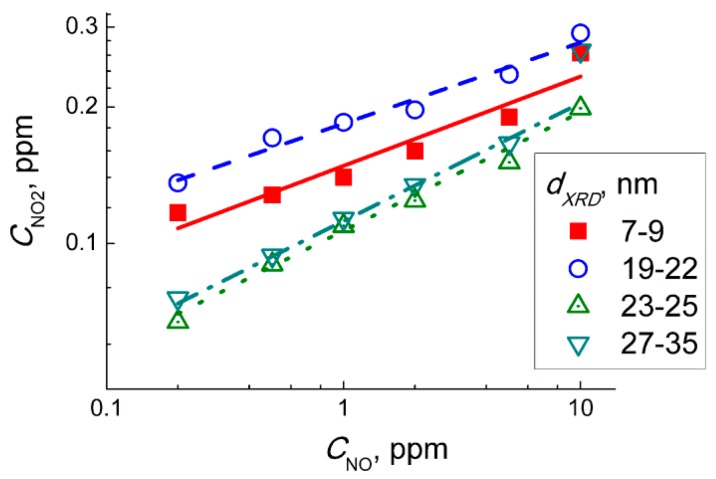
Concentration of NO_2_ in the gas mixture NO + air estimated from the sensor signals of WO_3_ with different grain size to the given concentrations of NO, under the assumption that the latter were controlled by incipient NO_2_ formed by gas-phase oxidation of NO in air.

Moreover, the sensitivity of WO_3_ sensors to 1 ppm NO_2_ (Figure 7a) was one to two orders of magnitude higher than the sensitivity to 5 ppm NO (Figure 7b). This implies that even traces of NO_2_ yielded in the reaction (Equation (2)) could guide the responses of WO_3_ sensors exposed to NO + air gas mixture. To check this hypothesis, we analyzed the dependencies of sensor signals on concentrations of target gases at 100 °C (Figure 6a,b). The sensor signals fitted to a power-law dependent on the concentration of NO_2_ (inset in Figure 6a) and NO (inset in Figure 6b), which is typical for porous sensing layers based on nanocrystalline *n*−type SMOx [31]. Assuming that the sensor signals to NO were masked by those to incipient NO_2_, i.e., *S*(NO) ≈ *S*(NO_2_ from NO oxidation), we estimated the concentration of NO_2_ in the gas flow of NO + air using the dependence of the sensor signal on NO_2_ concentration (inset in Figure 6a). Figure 11 shows the plots of the relationship between the estimated incipient NO_2_ concentration and the concentration of NO in the target gas mixtures NO + air. The plots could be linearized in the logarithmic axes implying the relation:

C(NO_2_) ~ *C*(NO)^α^. (9)

The power α in Equation (9) was in the range α = 0.18–0.22 for WO_3_ sensors with different particle size. However, Equation (2) is of the second order by NO concentration [32], according to mass action law:

d*C*(NO_2_)/d*t* = *k* × *C*(NO)^2^ × *C*(O_2_). (10)

Hence, at a fixed time period (flowrate of target gas and the volume of measuring setup were constant) the concentration of incipient NO_2_ should be proportional to square NO concentration (α = 2). Thus, from the inconsistence of experimental and theoretical values of the parameter α, we conclude that the response of WO_3_ sensors to NO was not controlled by NO_2_ from the gas-phase oxidation reaction (Equation (8)).

However, the occurrence of NO_2_ in gas mixture NO + air due to gas-phase oxidation was evidenced by the similar DRIFT spectral changes observed on exposure to NO_2_ (Figure 9) and NO (Figure 10). At room temperature, the NO adsorption on WO_3_ yielded similar surface species (Figure 10a), as did the adsorption of NO_2_ (Figure 9a). The DRIFT spectra of WO_3_ interacting with NO at 100 °C in dry air (Figure 10b) resembled those under the exposure to NO_2_, except for the case of WO_3_ with the smallest particle size (Figure 9b). It is likely that NO was, to some extent, oxidized in the gas phase, according to Equation (8), and the interaction with resultant NO_2_ proceeded as expressed in Equations (2)–(6). The extent of NO conversion to NO_2_ could be estimated to the order of 10%, since the evolution of peaks with comparable intensities, as seen in Figure 9 and Figure 10, were caused by the reaction with 2 ppm NO_2_ and with a 10-fold higher concentration of NO (20 ppm), respectively. Notably, the difference between sensor signals to 5 ppm NO (Figure 7b) and 1 ppm NO_2_ (Figure 7a) exceeded the order of 10%, which opposes the attribution of NO sensing route to gas-phase oxidation (Equation (8)) followed by the interaction of WO_3_ with incipient NO_2_ (Equations (2)–(6)). However, the main difference was found for the effect of humidity on the interaction with NO (Figure 10c). Suppressed evolution of nitro groups and the disappearance of the nitrate species suggest that humidity prevents the adsorption of NO_2_ resultant from the gas-phase NO oxidation, when the latter was the main component of the target gas. The contrast to the case of interaction with NO_2_ + H_2_O may be due to the fact that NO is not an acid-related oxide and it is not reactive to water, as in Equation (7).

Thus, based on DRIFT study in dry air, the interaction of WO_3_ with NO might be tangled by the reaction with NO_2_ originated from gas−phase NO oxidation in air (Equation (8)). However, the concentrations of NO_2_ approximated from the sensor signals mismatch those of NO in air (Figure 11), and the distinct effects of humidity were found on the DRIFT spectra of WO_3_ interacting with two nitrogen oxides. It prevents adjusting the NO sensing route to that of NO_2_ (Equations (2)–(6)) preceded by NO oxidation (Equation (8)). To account for the increase of sensors resistance and the evolution of nitro species (NO_2_^−^) in the DRIFT spectra (Figure 10b,c), we assume that NO sensing is determined by the reaction on the surface of WO_3_ as follows:

NO_(gas)_ + ½ O_2(gas)_ + *e*^−^ = NO_2_^−^_(ads)_. (11)

The further transformations of nitro groups on WO_3_ surface could proceed according to Equations (7) and (9) that account for the occurrence of nitrate NO_3_^−^ and probably NO^+^ species, as well as W-O bond formation when WO_3_ was exposed to NO. In humid air, the reaction with NO (Equation (11)) may be deteriorated by the competitive water adsorption, as follows from the decrease of the bands on the oxidized nitrogen species and W-O bonds in DRIFT spectra (Figure 10c). The difference with the gas-phase oxidation (Equation (8)) is that in reaction (Equation (11)) the role of WO_3_ surface is significant (adsorption sites, W^5+^ donor sites), and instead of gaseous O_2_ it can involve chemisorbed oxygen. In such a case, the sensing route of NO can be subtly dependent on the bulk and surface properties of a solid and on the sensing test conditions. This can account for the disagreement between the proposed sensing routes and even in attributing NO to the oxidizing or reducing gases, when the latter was detected by different SMOx as reported in [12,13,14,20,21,22,23,24,25,26]. The lower sensitivity of WO_3_ sensors to NO, in comparison to NO_2_, should therefore be due to distinct initial steps in the sensing routes. In the reaction with NO_2,_ it was the one-electron reduction (Equation (3)) favored by strong oxidizing activity of nitrogen dioxide and the presence of donor sites in WO_3_ (W^5+^, oxygen vacancies). The interaction with NO (Equation (11)) is essentially the oxidation of the target gas mediated by oxygen on the surface of WO_3_. This should be the main reason for different sensitivity to NO_2_ and NO, although the surface species formed in both interaction routes are similar. The overall reaction (Equation (11)) might proceed through different steps. For example, NO reduction to NO^−^, as proposed in [2,3,6,22,23,25], but not supported by the presently reported DRIFT study. Hence, the assumingly formed NO^−^ anions should be immediately oxidized by oxygen to NO_2_^−^ and NO_3_^−^ species. Further research of nitric oxide adsorption and sensing by tungsten oxide in oxygen-lean and inert media may be valuable to verify the proposed sensing mechanism.

## 5. Conclusions

Nanocrystalline WO_3_ with a grain size ranging from 7–9 nm to 27–35 nm was obtained by aqueous deposition of tungstic acid and heat treatment. The element composition and oxidation states were studied by XPS and EPR spectroscopy. The W^5+^ sites were observed by both techniques and the estimated concentration of W^5+^ was higher as the WO_3_ particle size decreased. The sensors based on WO_3_ demonstrated the responses to NO_2_ and NO as to oxidizing gases with the maximum sensitivity at 100 °C. The sensor signals to NO_2_ were higher than to NO, which should be due to the higher electron affinity and oxidation activity of the former. The WO_3_ sensors had at least 10 times lower cross-sensitivity to interfering reducing gases CO, NH_3_, H_2_S, and CH_4_. The sensor signals to NO_2_ and NO increased with a decrease of WO_3_ particle size, suggesting the influence of increasing concentration of donor sites W^5+^ in sensing nitrogen oxides. In situ infrared spectroscopic study (DRIFT), showed that the exposure of WO_3_ to NO_2_ and NO results in similar spectral features: decreased free-charge carrier concentration; surface oxidation of WO_3_; evolution of NO_2_^−^; and adsorbed NO_2_, NO_3_^−^ and probably NO^+^. However, some differences were found in the effect of humidity on the evolution of the oxidized nitrogen species on the surface of WO_3_ during the interaction with NO_2_ and NO. From the analysis of the dependencies of WO_3_ sensor signals on the concentrations of target gases, it was inferred that the NO sensing mechanism was not controlled by NO_2_, as could be expected from thermodynamically favorable gas-phase NO oxidation in air. The routes of NO_2_ and NO interactions with WO_3_ were proposed that account for the similar, but independent, sensing routes.

## Supplementary Materials

The following are available online at https://www.mdpi.com/1424-8220/19/15/3405/s1, Figure S1: Dynamic sensors responses to 1 ppm NO_2_ at different temperature 300–25 °C; Figure S2: Dynamic sensors responses to 5 ppm NO; Figure S3: Dynamic sensors responses to 1 ppm NO_2_ at 100 °C and different relative humidity 0–90% r.h.; Figure S4: Dynamic sensors responses to 5 ppm NO at 100 °C and different relative humidity 0–90% r.h.; Figure S5: Comparison of sensor signals of WO_3_ with different grain size at 100 °С to 1 ppm NO_2_, 5 ppm NO, 20 ppm CO, 20 ppm NH_3_, 2 ppm H_2_S, and 100 ppm CH4. Figure S6: Sensor signals of WO_3_ sensor with grain size 7–9 nm to 1 ppm NO_2_ and 5 ppm NO measured on different dates during 2 months.
